# COVID-19 in HIV: a Review of Published Case Reports

**DOI:** 10.1007/s42399-020-00593-6

**Published:** 2020-11-02

**Authors:** Zoya Morani, Saumil Patel, Sudeshna Ghosh, Falah Abu Hassan, Shriya Doreswamy, Sandeep Singh, Venkata Neelima Kothapudi, Rupak Desai

**Affiliations:** 1 Washington University of Health and Science, San Pedro, Belize; 2 Carefirst Primary and Wellness Center, Dallas, TX USA; 3 UT South Western Medical Center, Dallas, TX USA; 4 Princess of Wales Hospital, Bridgend, UK; 5 Vydehi Institute of Medical Sciences and Research Center, Bangalore, India; 6 Department of Clinical Epidemiology, Biostatistics and Bioinformatics, Amsterdam University Medical Center, AMC, Amsterdam, Netherlands; 7 Department of Medicine, Gandhi Medical College, Secunderabad, Telangana India; 8 Division of Cardiology, Atlanta VA Medical Center, Decatur, GA USA

**Keywords:** COVID-19, HIV, SARS-CoV-2

## Abstract

Patients with COVID-19 present with a myriad of comorbidities. An immunocompromised state like HIV in patients with COVID-19 can be life-threatening. We searched PubMed/Medline, Scopus, and Web of Science for case reports and case series about COVID-19 in HIV patients. We finally reviewed 20 case reports including cases of 43 patients with HIV and COVID-19. The mean age of 43 adult patients was 51.56 ± 27.56 years (range 24–76 years). Of these, 30 were male (69.77%), 11 were female (25.58%), and 2 were transgender (4.65%). A total of 25 patients (58.14%) were above 50 years of age. The most common cardiovascular comorbidities were hypertension and hyperlipidemia (48.8%), diabetes (20.93%), and morbid obesity (11.63%). Out of 43 HIV patients with COVID-19, 6 resulted in death (13.95%). All the patients who died were elderly above 50 years and required mechanical ventilation. HIV patients infected with COVID-19 had a high mortality rate. A high burden of pre-existing comorbidities and an advanced age in these patients make them prone to disease progression and worse outcomes.

## Introduction

Coronavirus disease (COVID-19), caused by the novel SARS-CoV-2 virus, was declared a global pandemic by the World Health Organization on 11 March 2020. Earlier it has caused diseases like Middle East respiratory syndrome (MERS) in Saudi Arabia in 2012 and severe acute respiratory syndromes (SARS) in China in 2002. The single-stranded RNA genome virus has overwhelmed the healthcare system all across the world. People living with human immunodeficiency virus (HIV), advanced age (> 60 years), hypertension (HTN), and diabetes mellitus (DM) are at an increased risk of mortality and morbidity. HIV (human immunodeficiency virus) pandemic coexisting with another pandemic like COVID-19 is called syndemic. This group of HIV-positive individuals, who are unable to practice socialdistancing, have limited access to healthcare, and are prone to drug abuse, will be severely affected. The antiretroviral therapy (ART) has helped in prolonging the lifespan of HIV-positive patients, but the concurrent occurrence of COVID-19 and HIV is presenting unique challenges to the management of these patients. Difficulty in the timely refill of ART medications, a restricted visit to doctors, increased stressors like loneliness, loss of jobs, and fear of homelessness could culminate in substance abuse, a rise in acquired immunodeficiency syndrome (AIDS)–related diseases, and psychiatric problems [1]. The condition could be worse in areas with the already overburdened healthcare system and diversion of public health resources to combat COVID-19. It can make this vulnerable population face unprecedented challenges to maintain their continuity of care [2]. With no cure at hand, practicing social distancing is playing a major role in minimizing the spread of the coronavirus infection but can result in decreased adherence to ART therapy and the health outcomes of these patients will take a downhill course [3]. Therefore, we aim to systematically review all published cases of HIV patients with COVID-19 and related clinical correlates and outcomes.

## Methods

We searched PubMed/Medline, Web of Science, and Scopus until 1 August 2020 for case reports and case series using the following keywords: COVID-19, SARS-CoV-2, HIV, and human immunodeficiency virus. All published case reports included in the final analysis were in the English language. Our search identified 167 studies in total. After removing the duplicates and selecting case reports or case series with individual patient-level data, we found 23 articles. Due to a lack of data, we further excluded 3 articles and finally include 20 articles [4–23]. Continuous variables were presented as means ± standard deviations and categorical data as absolute values and percentages. All data extraction and descriptive analysis were performed using Microsoft Excel.

## Results

We identified 23 articles through our search from which we excluded 3 due to lack of data. Using 20 articles we selected, a total of 43 patients were analyzed. The mean age of the patients was 51.56 ± 27.56 (range 24–76 years). Of the 43 patients, 30 were male (69.77%), 11 were female (25.58%), and 2 were transgender (4.65%). The race was not included in the analysis due to it not being reported in the data. The majority of the cases were from the USA (58.14%), followed by China (13.95%), the UK (6.98%), Iran (4.65%), South Korea (4.65%), Austria (2.33%), Cyprus (2.33%), Italy (2.33%), Japan (2.33%), and Singapore (2.33%) (Table 1). A total of 25 patients had cardiovascular/pulmonary comorbidities (58.14%), the most predominant one being HTN in 14 patients (32.56%) out of which less than half had hyperlipidemia (HLD). Of 44 patients, 9 had diabetes mellitus (20.93%), 5 had the chronic obstructive pulmonary disease (11.63%), and 3 patients had a history of atrial fibrillation (6.98%).

**Table 1:** Demographics, comorbidities, and presentation of COVID-19 in HIV patients

Author/year	Age (years)/sex (M/F)	Country	Past medical history	Cardiovascular/pulmonary comorbidities	Contact history	Presenting symptoms	Meantime from exposure to infection
Jin Sun et al./2020	37/M	Singapore	None	None	NA	Fever, sore throat, dry cough, and headache	NA
Toombs et al./2020	62/M	UK	Renal transplant 2012 T2DM on insulin Hypertension Latent tuberculosis	HTN	NA	Dyspnea and a dry cough	NA
Toombs et al./2020	46/M	UK	Smoker G6PD deficiency	None	NA	Productive cough and fevers	NA
Toombs et al./2020	57/F	UK	Hypertension T2DM Obesity Stroke 2007 Graves’ disease—in remission Reflux	HTN	NA	Dyspnea, a dry cough, fevers, anorexia, and headaches	NA
Menghua et al./2020	49/F	China	Cured syphilis and viral pneumonia	None	NA	Fever, pharyngeal pain, and chills	NA
Giambenedetto et al./2020	75/M	Italy?	HIV, hep B	HTN	NA	High fever, diarrhea, and cough	NA
Choi et al./2020	71/M	South Korea	None	HT	NA	Fever and cough	NA
Choi et al./2020	67/F	South Korea	None	None	NA	Fever and myalgia	NA
Müller et al. 2020	55/M	Austria	HCV, HCC, cirrhosis, and liver transplant	None	A friend with flu symptom	Fever, fatigue, and tachycardia	12 days
Ali asadollahi-amin et al./2020	44/M	Iran	Rib fracture	None	NA	Chest pain and local tenderness	NA
Ridgway et al./2020	38/M	USA	None	DM, HTN, OSA, and obesity	NA	Headache, myalgia, SOB, fever, diarrhea, and tachycardia	NA
Ridgway et al./2020	50/F	USA	None	Obesity	NA	Fever, cough, SOB, and headache	NA
Ridgway et al./2020	51/F	USA	Latent tuberculosis	None	NA	Fever, cough, SOB, and diarrhea	NA
Ridgway et al./2020	53/F	USA	Esophageal strictures and bronchoesophageal and tracheoesophageal fistulas	None	Denied sick contacts	Fever, cough, vomiting, and diarrhea	NA
Ridgway et al./2020	47/F	USA	HF (LVEF-15%), ICD, PE, and CVA	HTN, obesity, and COPD	Denied sick contacts	Chest pain, SOB, tachycardia, abdominal pain, and diarrhea	NA
Haddad et al./2020	41/M	USA	Recurrent HSV	None	Indirect exposure to a COVID-19-positive patient	Abdominal pain, vomiting, dry cough, intermittent fever, and confusion	NA
Zhu et al./2020	61/M	China	DM Chronic smoker	None	NA	Fever and dry cough	NA
Chen et al./2020	24/M	China	None	None	NA	Fever and dry cough	NA
Wu et al./2020	61/M	China	Pulmonary TB and DM	None	NA	Generalized myalgia, intermittent fever, fatigue, sob, and productive cough	NA
Wu et al./2020	47/M	China	None	None	NA	Fever, generalized myalgia, sore throat, cough, intermittent shortness of breath, and diarrhea	NA
Patel et al./2020	58/M	USA	None	None	NA	Weakness, anorexia, and diarrhea	NA
Iordanou et al./2020	58/M	Cyprus	Influenza A and B	None	NA	Dry cough, malaise, and fever	NA
Sadr et al./2020	57/F	Iran	None	None	A pharmacist exposed to COVID-19 patients	Headache, malaise, and fever	NA
Wang et al./2020	37/F	China	None	None	NA	Fever, dry cough, chest hypoxia, tachypnea, high BP, and tachycardia	NA
Benkovic et al./2020	56/M	USA	None	HLD	Trip to Florida	Fatigue anosmia ageusia	NA
Benkovic et al./2020	56/M	USA	None	HTN	NA	Fever and fatigue	NA
Benkovic et al./2020	62/M	USA	HCV	HTN HLD	NA	Fever, nonproductive cough, fatigue, and watery diarrhea	NA
Benkovic et al./2020	65/M	USA	AFIB, DM	HTN HLD	NA	Cough, fever, and hypoxia	NA
Blanco et al./2020	40/transgender	USA	None	None	NA	URTI, fever, cough, malaise, headache, and high BP	NA
Blanco et al./2020	49/M	USA	Hypothyroidism	None	HCW	LRTI, fever, and cough PaO_2_–182	NA
Blanco et al./2020	29/M	USA	None	None	Sexual worker participant in chemsex session	URTI-fever, cough, malaise, and headache	NA
Blanco et al./2020	40/M	USA	Asthma	None	Dinner 5 days before with another person who was COVID-19 positive	Lower respiratory tract infection-fever, cough, malaise, headache, dyspnea, and tachycardia	NA
Blanco et al./2020	31/transgender	USA	None	None	NA	Lower respiratory tract infection-fever, cough, dyspnea, and tachycardia	NA
Suwanwongse et al./2020	37/M	USA	Tertiary syphilis HCV	None	NA	Cough Myalgia Rhinorrhea \	NA
Suwanwongse et al./2020	31/M	USA	None	Obesity and HLD	NA	Dyspnea, cough, fever, and tachycardia	NA
Suwanwongse et al./2020	70/M	USA	AFIB, HF, HCV, and COPD	HTN and HLD	NA	Dyspnea, tachycardia, and tachypnea	NA
Suwanwongse et al./2020	76/F	USA	DM	None	NA	Cough and fever	NA
Suwanwongse et al./2020	63/M	USA	HTN	None	NA	Watery diarrhea, vomiting, and fever	NA
Suwanwongse et al./2020	52/M	USA	DM	None	NA	Dyspnea, tachycardia, and tachypnea	NA
Suwanwongse et al./2020	58/M	USA	DM	HTN, HLD, and COPD	NA	Fever, cough, dyspnea, tachycardia, and tachypnea	NA
Suwanwongse et al./2020	52/M	USA	HCV	HTN, HLD, and COPD	NA	Fever, cough, dyspnea, tachycardia, and tachypnea	NA
Suwanwongse et al./2020	76/F	USA	AFIB	HTN, pulmonary HTN, and COPD	NA	Fever, cough, dyspnea, and tachypnea	NA
Nakamoto et al./2020	28/M	Japan	Smoking, alcohol drinking, and HBV (+)	None	NA	Pneumonia	NA

*HTN*, hypertension; *DM*, diabetes mellitus; *HSV*, herpesvirus; *NHL*, non-Hodgkin’s lymphoma; *AFIB*, atrial fibrillation; *HID*, hyperlipidemia; *COPD*, chronic obstructive pulmonary disease; *HCV*, hepatitis C virus; *URTI/LRTI*, upper/lower respiratory infection; *HBV*, hepatitis B virus; *HF*, heart failure

In terms of presenting symptoms, out of 43 patients, 32 presented with fever (74.42%), 29 presented with cough (67.44%), 9 presented with diarrhea (20.93%), 8 reported headaches (18.60%), 11 had tachycardia (25.58%), 16 had shortness of breath/dyspnea (37.21%), 2 patients had hypoxia (4.65%), 1 reported pneumonia (2.33%), 5 patients presented with fatigue (11.63%), 2 presented with vomiting (4.65%), 1 reported weakness (2.33%), 5 reported myalgia (11.63%), 1 other patient reported anosmia and ageusia (2.33%), and a patient also presented with a sore throat. Of 32 patients who presented with fever, 2 had an upper respiratory tract infection and 3 had a lower respiratory tract infection. Out of the 29 (85.29%) patients presenting with cough, 8 reported having a dry cough.

When looking at the contact history of the 43 patients, 34 patients (79.07%) had no contact history reported. Out of the 9 patients who did have a contact history, 2 denied any sick contacts, 1 had a friend with flu symptoms, 1 had an indirect exposure to a COVID-19-positive patient, 1 of the patients was a pharmacist exposed to COVID-19, another was a sex worker, 1 had dinner 5 days prior with a COVID-19-positive person, another took a trip to Florida, and lastly 1 of the exposed patients was a healthcare worker. There was only one case that reported the meantime from exposure to infection of 12 days. CD4 counts were reported in 32 patients of which, 17 (53.13%) had a CD4 count lower than the normal range (< 500 cells/mm^3^). Nine (20.93%) of the patients had undetectable HIV-RNA (copies/ml) or viral load. Of 30 cases reporting SPO_2_ data, 13 (45.1%) had oxygen saturation less than 95% at the time of admission.

Out of 43 HIV patients with COVID-19, 6 (13.95%) patients died during hospitalization, while the remaining 37 (86.05%) recovered (Table 2). The mean age of the patients that resulted in death was 63.5 ± 12.5 (range 52–76). Of the 6 that died, 5 had bilateral ground-glass opacities on computed tomography scan/chest x-ray, had low oxygen saturation, required mechanical ventilation, and their hospitalization ranged from 1 to 14 days. Of the 37 recovered, 8 patients were on hydroxychloroquine (HCQ) and 1 patient was on chloroquine (CQ). All the patients were on different antiretroviral therapy regimens.

**Table 2:** Diagnostics, laboratory investigations, and outcomes of COVID-19 in HIV patients

Author/year	Age/sex	COVID test	Chest imaging	CT imaging	Rx COVID	Rx HIV	Rx of comorbidity	Mechanical ventilation (intubation)	Hospital stay (days)	Outcome
Jin Sun et al./2020	37/M	rRT-PCR	Clear with no infiltrates or consolidation	NA	None due to mild illness No treatment	TFV, 3TC RPV	NA	NA	14	Recovery
Toombs et al./2020	62/M	RT-PCR	Bilateral opacities	NA	TZP AZM TMP-SMX PRED	RAL 3TC ABC TAC mycophenolate	NA	Yes	8	Death
Toombs et al./2020	46/M	RT-PCR	-	NA	LVX Atovaquone PRED	TDF/FTC DTG	NA	Yes	6	Recovered
Toombs et al./2020	57/F	RT-PCR	Bilateral consolidation	NA	DOX TMP-SMX	TAF/FTC NVP	NA	Yes	10	Recovered
Menghua et al./2020	49/F	RT-PCR	NA	Ground-glass dense shadow and cord shadow under the pleura of the lateral segment of the middle lobe and a dorsal-base segment of the lower lobe of the right lung	CXM traditional Chinese medicine (Lian-qin oral solution and LianhuaQingwen capsule) Then changed to interferon atomization, ribavirin, Arbidol, and moxifloxacin	EFV ZDV 3TC	NA	Yes	47	Recovered
Giambenedetto et al./2020	75/M	RT-PCR	Bilateral signs of interstitial pneumonia Ground-glass opacity in the anterior segment of the RU lobe	Bilateral consolidationsand ground-glass opacities	HCQ, AZM = discount later due to cardiotoxicity and conduction disorder Sarilumab IV = recovery after this was administered and HCQ and AZM were discontinued	Darunavir, COB FTC TAF	Perindopril for HTN	Yes	19	Recovery
Choi et al./2020	71/M	rRT-PCR	Mild opacity in RLL>>rapidly aggravated bilateral infiltration	NA	HCQ, MPD, day, convalescent plasma, and O_2_ via a nasal prong	LPV/r	NA	Yes	26	Recovery
Choi et al./2020	67/M	rRT-PCR	LLL infiltration	NA	HCQ, MPD, convalescent plasma, and oxygen	LPV/r	NA	Yes	24	Recovery
Mülleret al./2020	55/M	PCR	Diffuse bilateral infiltration	NA	Antibiotics, immunosuppressive, and oxygen via nasal prongs	FTCTAF RPV	Hemophilia A-factor VIII, HCV-IFN, tacrolimus, mycophenolate, and steroids-2019	No	6	Recovery
Asadollahi-Amin et al./2020	44/M	rRT-PCR	NA	Patchy ground-glass opacityin the upper lobeof the right lung	HCQOTV	LPV/r	NA	No	5	Recovery
Ridgway et al./2020	38/M	SARS-CoV-2 PCR	Perihilar patchy opacities	Bilateral ground-glassopacities	CRO AZM HCQ	ABC DTG 3TC	NA	No	5	Recovery
Ridgway et al./2020	50/F	SARS-CoV-2 PCR	Mild multi-focal patchy airspace consolidation in the left lower lobe	NA	Oxygen via nasal prongs, AZM, CRO, CDR	BIC FTC TAF	NA	No	4	Recovery
Ridgway et al./2020	51/F	SARS-CoV-2 PCR	Bilateral perihilar and basilar patchy airspace and interstitial opacities	NA	CRO AZM CDR HCQ	ART regimen of elvitegravir, COB FTCand TAF (missed 5 days)	NA	No	7	Recovery
Ridgway et al./2020	53/F	SARS-CoV-2 PCR	Unremarkable	NA	CDR AZM	BIC FTC TAF RTV DRV TDF FTC	NA	No	3	Recovery
Ridgway et al./2020	47/M	SARS-CoV-2 PCR negative on admission, positive on day 3	Cardiomegaly but no infiltrate	Wedge-shapedsplenic infarction	NA. Self-discharged against advice on day 3	TDF, FTC DRV RTV RAL	NA	No	2	Recovered
Haddad et al./2020	41/M	SARS-CoV-2 PCR negative on admission, positive on day 3 (COVID test positive)	NA	Diffuse patchy nodular, ground-glass infiltrates	HCQ, AZM, FEP AMP VAN	DTG-3TC	Cefepime, ampicillin, vancomycin, and acyclovir for empiric, bacterial meningitis and herpes encephalitis coverage	Yes	6	Recovery
Zhu et al./2020	61/M	rRT-PCR	NA	Pneumonia withfindings of multiple ground-glass opacities(GGO) in bilateral lungs>>>progressiveGGO and consolidationin lungs	MXF γ-globulin MDP and O_2_ via nasal prongs	LPV/r	Alogliptin co-administered with metformin	No	21	Recovery
Chen et al./2020	24/M	rRT-PCR	NA	Multiple high-density patchy shadows with unclear boundaries in the subpleural regions of the middle and lower lobes of the right lung, with the involvement of adjacent interlobar pleura	Interferon inhalation	LPV/r TFV 3TC EFV	NA	No	15	Recovery
Wu et al./2020	60/M	rRT-PCR	NA	Bilateral multiple ground-glass opacities (GGO), prominenton the right lower lobe	Oxygen OTV MXP CRO and tazobactam)	TDF 3TC EFV	CHOP and EPOC-B cell lymphoma TB-INH, rifabutin, ethambutol and moxifloxacin, insulin-DM	No	14	Recovery
Wu et al./2020	47/M	rRT-PCR	NA	Bilateral multiple GGO	Oxygen, MXF RBV umifenovir, YZP SMZ	NA	NA	No	25	Recovery
Patel et al./2020	58/M	rRT-PCR	Clear lungs	NA	,HCQ AZM, and zinc sulfate	FTC TFV ATVRTV	NA	No	5	Recovery
Iordanou et al./2020	58/M	rRT-PCR (positive on third swab test on day 6)	Bilateral air space pacifications	NA	LVX OTV AZM TZP VAN MEM GEN CAS enoxaparin	EVG COB FTC TDF	NA	Yes	32	Recovery
Sadr et al./2020	57/F	RT-PCR	Unremarkable	Unremarkable	NA	NA	NA	No	7	Recovery
Wang et al./2020	37/M	RT-PCR negative four times. SARS-Cov-2 IgM was positive		Multipleinfiltrations inboth lungs	Umifenovir, CS Human serum albumin, thymosin, and ulinastatin Tocilizumab High-flow oxygen (15 l/min)	NA	NA	No	NA	Recovery
Benkovic et al./2020	56/M	rRT-PCR	NA	NA	NA	FTC TFV etravirine, ABC	HTN-lisinopril	No	Home isolation	Recovery
Benkovic et al./2020	56/male	RT-PCR	Pneumonia	NA	NA	FTCTFV TAF	Rosuvastatin and losartan	No	Home isolation	Recovery
Benkovic et al./2020	62/M	RT-PCR	NA	NA	NA	FTCEVG COB TAF	HTN-losartan, DM-metformin, HLD-atorvastatin, and AFIB-Coumadin	No	14	Recovery
Benkovic et al./2020	65/M	RT-PCR	NA	NA	NA	TAF FTC DRV-boosted COB	NA	No	1	Recovery
Blanco et al./2020	40/transgender	PCR	Normal	NA	NA	TAF FTC DRV-boosted COB	NA	No	1	Recovery
Blanco et al./2020	49/M	PCR	Bilateral ground-glass opacities	NA	Interferon beta-1b, HCQ, MEM LZD tocilizumab	ABC 3TC DTG TDF FTC LPV/r	NA	Yes	21	Recovery
Blanco et al./2020	29/M	PCR	NA	NA	HCQ, AZM	TAF FTC DRV-boosted COB TDF FTC LPV/r	NA	No	3	Recovery
Blanco et al./2020	40/M	PCR	Right basal interstitial infiltrates	NA	AZM, CFM HCQ, inhaled corticosteroids	ABC 3TC DTG TDF FTC LPV/r	NA	No	4	Recovery
Blanco et al./2020	31/transgender	PCR	Right basal pneumonia with pleural effusion	NA	Interferon beta-1b and HCQ, AZM, CFT TMP-SMX, and corticosteroids	TAF FTC DRV-boosted COB	NA	No	12	Recovery
Suwanwongse et al./2020	37/M	RT-PCR	Normal	NA	Symp Trt. No antibiotics	FTC, TAF, and DTG	NA	No	1	Recovery
Suwanwongse et al./2020	31/M	RT-PCR	Bilateral multifocal infiltrates	NA	Symp Trt. No antibiotics	EVG, FTC, TAF, and COB-compliant	NA	No	3	Recovery
Suwanwongse et al./2020	70/M	RT-PCR	Bilateral ground-glass opacities	NA	HCQ, AZM CF3	FTC, TDF, and RAL-compliant	NA	Yes	12	Death
Suwanwongse et al./2020	76/ F	RT-PCR	Bilateral ground-glass opacities	NA	HCQ CF3	FTC, TAF, ATV, and COB-compliant	NA	Yes	7	Death
Suwanwongse et al./2020	63/M	RT-PCR	Bilateral ground-glass opacities	NA	HCQ AZM CF3	FTC, TAF, and DTG-compliant	NA	Yes	13	Death
Suwanwongse et al./2020	52/M	RT-PCR	Bilateral ground-glass opacities	NA	HCQ	EVG, FTC, TAF, and COB-non compliant	NA	Yes	1	Death
Suwanwongse et al./2020	58/M	RT-PCR	Bilateral interstitial infiltrates	NA	HCQ AZM CF3	HARRT-not taking	NA	Yes	14	Death
Suwanwongse et al./2020	52/M	RT-PCR	Bilateral multifocal infiltrates	NA	COVID treatment-none Antibiotic-tazocin and doxycycline	FTC, TDF, and DTG-compliant	NA	No	3	Recovery
Suwanwongse et al./2020	76/F	RT-PCR	Bilateral multifocal infiltrates	NA	AZM	EFV, FTC, and TAF-compliant	NA	No	5	Recovery
Nakamoto et al./2020	28/M	Not mentioned	NA	Multiple GGO	HCQ	NA	NA	No	8	Recovery

*ABC*, abacavir; *ACV, *acyclovir; *AMP, *ampicillin; *ATV, *atazanavir; *AZM,* azithromycin; *BIC*, bictegravir; *CAS*, caspofungin; *CDR, *cefdinir; *FEP*, cefepime; *CFM*, cefixime; *CFP*, cefoperazone; *CRO*, ceftriaxone; *CXM*, cefuroxime; *CPT*, ceftaroline; *CF3*, 3rd generation cephalosporins; *COB*, cobicistat; *DRV*, darunavir; *DTG*, dolutegravir; *DOX*, doxycycline; *EFV*, efavirenz; *EVG*, elvitegravir; *FTC*, emtricitabine; *EMB*, ethambutol; *ETR*, etravirine; *GEN*, gentamicin; *HCQ*, hydroxychloroquine; *LZD*, linezolid; *LPV/r*, lopinavir/ritonavir; *MEM*, meropenem; *MXF*, moxifloxacin; *NVP*, nevirapine; *OTV*, oseltamivir; *SUL*, sulbactam; *TZP*, piperacillin/tazobactam(tazocin); *Pred*, prednisone; *RAL*, raltegravir; *RFB*, rifabutin; *RPV*, rilpivirine; *RTV*, ritonavir; *3TC*, lamivudine; *LVX*, levofloxacin; *RBV*, ribavirin; *TAC*, tacrolimus; *TAF*, tenofovir alafenamide; *TFV*, tenofovir; *TDF*, tenofovir disoproxil fumarate; *TAF/FTC*, tenovovir alafenamideemtricitabine (Descovy); *TDF/FTC*, tenovovir disoproxil fumarate/emtricitabine (Truvada); *TMP/SMX*, trimethoprim/sulfamethoxazole (co-trimoxazole); *VAN*, vancomycin; *ZDV*, zidovudin; *NA*, not available; *Symp*, symptomatic; *Trt*, treatment; *GGO*, ground glass opacity; *RT-PCR*, reverse transcriptase-Polymerase Chain Reaction

## Discussion

The key findings of the current review suggest that HIV patients with COVID-19 infection have a high burden of cardiovascular comorbidities. Furthermore, most of the patients were elderly and male. The most common presenting symptoms were fever, cough, and shortness of breath as reported in COVID-19 patients. Besides, the majority of HIV patients with COVID-19 infection were on ART therapy. The patients who did not recover were mostly elderly (> 50 years) and had cardiovascular comorbidities including HTN, DM, or both. A study by Shahid et al. stated that the older COVID-19 patients with comorbidities such as DM have an increased risk of mortality [24]. Of 6 patients who died, 5 received HCQ with some having a combination of HCQ and/or azithromycin (AZM) and/or 3rd-generation cephalosporins. Of the 37 recovered patients, 11 were on ritonavir (RTV), and 8 of which were on lopinavir (LPV) as well. A study by Yu and colleagues found that influenza-coinfected patients taking lopinavir/ritonavir(LPV/r) treatment had faster pneumonia recovery than those who did not [25].

A therapeutic protective role of anti-HIV agents against COVID-19 infections has been reported [15]. Besides this, high mortality (13.95%) was reported in these patients. Of those that recovered, 17 (39.53%) were reported to be taking a combination of tenofovir (TDF) and emtricitabine (FTC) and 3 (6.98%) patients were taking TDF with other combinations of drugs. A study by Amo et al. suggested that HIV-positive patients on treatment with TDF and FTC proved to have a lower risk of COVID-19-related hospitalization [26]. Only 1 out of the 6 patients that did not recover was receiving tenofovir (TDF) and emtricitabine (FTC) as a combination. ART therapy seems to play a crucial role in protecting HIV patients from COVID-19-related hospitalization. However, key challenges have been reported by this high-risk population in timely accessing pre- and post-exposure prophylaxis during this pandemic [27]. Policymakers in different countries have proposed and implemented a support framework at a different level to support HIV patients during the COVID-19 pandemic [27, 28].

## Limitations

Limitations associated with this article should be taken into due consideration while drawing any inference. This review only included the case reports or case series with individual patient-level data so the finding could not be generalized. Furthermore, owing to the small sample size, it is not possible to compare the findings between the deceased and survived groups. Besides, data on CD4 count and viral load were missing in a lot of patients which would make it difficult for any subgroup comparison.

## Conclusion

In our review, we found that HIV patients with COVID-19 had a high burden of HTN and/or DM and were over the age of 50. The patients who recovered were on a combination of specific ART therapy which was backed up by research in having a protective role against COVID-19 which could have played a role in their recovery. This review gives a glimpse to look deeper into other treatment options such as anti-viral agents like TDF and LPV/r given that the patients over the age of 50 years with HTN and/or DM that did end up recovering were on one or another of these medications.
